# Ossifying pilomatricoma and a novel hypothesis for its pathogenesis: A case report and comprehensive literature review

**DOI:** 10.1097/MD.0000000000028753

**Published:** 2022-02-11

**Authors:** Kun-Yong Sung, Seungkoo Lee, Yeonjin Jeong, Sang-Yeul Lee

**Affiliations:** a Department of Plastic and Reconstructive Surgery, Kangwon National University School of Medicine, Chuncheon, Republic of Korea; b Department of Anatomic Pathology, Kangwon National University School of Medicine, Chuncheon, Republic of Korea; c Department of Plastic and Reconstructive Surgery, Kangwon National University Hospital, Chuncheon, Republic of Korea.

**Keywords:** foreign body reaction, heterotopic ossification, keratins, pilomatricoma, pilomatrixoma

## Abstract

**Rationale:**

Pilomatricoma is a benign skin appendageal tumor derived from hair follicle matrix cells that commonly affects the head, neck, and upper extremities of the pediatric population. Since the original tumor description, diverse variants have been reported in the literature. Pilomatricoma with florid osseous metaplasia is described as an ossifying pilomatricoma and is recognized as a distinct variant of this benign tumor. However, the pathogenesis of this variant remains unclear. In this study, we present an uncommon case of ossifying pilomatricoma and address the pathogenesis of metaplastic ossification through a comprehensive literature review.

**Patient concerns:**

A 14-year-old boy presented with an asymptomatic protuberant mass in the preauricular region.

**Diagnosis:**

Based on its clinicopathological features, we diagnosed the lesion as an ossifying pilomatricoma.

**Interventions and outcomes:**

The lesion was surgically removed under local anesthesia. The postoperative course was uneventful during the 6-month postoperative follow-up.

**Lessons:**

We suggest that metaplastic ossification in ossifying pilomatricoma represents another feature of foreign body reaction to keratinous materials containing shadow cells in old lesions and a walling-off phenomenon to prevent exposure of surrounding tissues to keratinous materials.

## 1 Introduction

Pilomatricoma, also known as calcifying epithelioma of Malherbe, is a benign skin appendageal tumor derived from hair follicle matrix cells. It usually manifests as an asymptomatic, firm subcutaneous nodule with bluish-red discoloration of the overlying skin and commonly occurs on the head, neck, and upper extremities of the pediatric population. Since Malherbe and Chenantais first described it in 1880 as a calcified tumor of the sebaceous glands, diverse variants have been reported in the literature, such as anetodermic/lymphangiectatic/bullous, aggressive, superficial, perforating, proliferating, ossifying, cystic, pseudocystic, pigmented, acantholytic, and malignant. They have similar origins but present different clinicopathological features that often pose a diagnostic dilemma for clinicians. Pilomatricoma with florid osseous metaplasia is referred to as an ossifying pilomatricoma and is recognized as a distinct variant of this benign tumor.^[1–4]^ The clinicopathological features of this variant have been well described in the literature, but the pathogenesis of metaplastic ossification remains poorly understood. In this report, we present an uncommon case of ossifying pilomatricoma originating from the preauricular region and address its pathogenesis through a comprehensive literature review.

## 2 Case report

**Figure 1:**
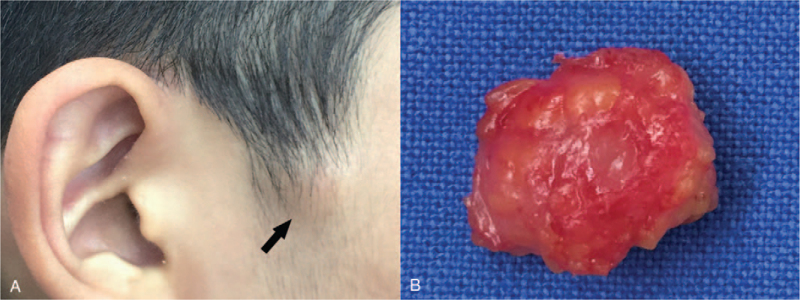
(A) A dome-shaped mass in the preauricular region (arrow). (B) A gross specimen shows a reddish-brown colored globular mass covered with fibroadipose tissue.

**Figure 2:**
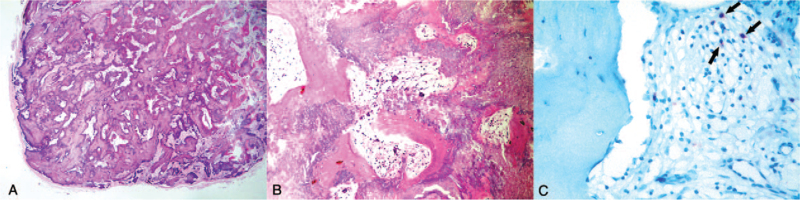
(A) Histopathological study shows a well-circumscribed nodular tumor with extensive ossification (H&E, 15×). (B) Histopathological study shows shadow cell clusters, laminated trabecular bones, osteoclast-like multinucleated giant cells, and bone marrow structures enclosed by bony trabeculae (H&E, 100×). (C) Immunohistochemical study shows a few cells (arrows) positive for antimyeloperoxidase antibodies within the bone marrow structure (400×).

A 14-year-old boy presented with a 5-year history of an asymptomatic protruding mass in the right preauricular region (Fig. 1A). Physical examination revealed a firm, dome-shaped mass (1.5 cm in diameter) adherent to the overlying skin without alteration of the skin. The entire mass was removed under local anesthesia. On gross examination, a reddish-brown globular mass with a stony hard consistency was noted (Fig. 1B). Microscopic examination showed a well-circumscribed nodular tumor with florid osseous metaplasia (Fig. 2A). The tumor was mainly comprised of shadow cell clusters and laminated trabecular bones with no basaloid components. The stroma admixed with shadow cell clusters showed extensive ossification along with osteoclast-like multinucleated giant cells. Bone marrow structures containing a few mononuclear cells and fibrofatty tissue were observed enclosed by bony trabeculae (Fig. 2B). On immunohistochemistry, these marrow cells were positive for antimyeloperoxidase antibodies (Fig. 2C). Based on the clinicopathological features, the lesion was diagnosed as an ossifying pilomatricoma. The postoperative course was uneventful at the 6-month follow-up.

## 3 Discussion

Histopathologically, pilomatricoma consists of 2 major cell types: basaloid and shadow cells. A conventional pilomatricoma shows a typical biphasic appearance with peripheral lobules of small, darkly stained basaloid cells maturing into larger, pink shadow cells in the center.^[5]^ Shadow cells, most likely to represent aberrant keratinization,^[6]^ trigger inflammatory and foreign body reactions when exposed to the surrounding stromal tissue because endogenous keratin is physiologically recognized as a foreign body. These cells provide important clues to follicular differentiation; however, they are not pathognomonic of pilomatricomas. Shadow cells are also observed in odontogenic tumors, craniopharyngiomas, and rarely in other lesions with follicular differentiation.

Pilomatricomas are categorized into 4 chronological stages: early, fully developed, early regressive, and late regressive.^[7]^ Early lesions are small cystic structures lined by squamoid and basaloid epithelial cells that contain faulty hair matrix material composed of shadow cells. In the fully developed stage, the faulty hair matrix material is released into adjacent tissues following rupture of the epithelial lining with rapid tumor growth, which attracts macrophages and elicits an inflammatory response. The early regressive stage exhibits granulation tissue with inflammatory infiltrate and multinucleated histiocytic giant cells around the shadow cells. The inflammatory infiltrate observed during the early regressive stage becomes sparse or completely disappears during the late regressive stage and is replaced by varying degrees of calcification, ossification, and desmoplastic stroma.

Ossifying pilomatricoma, characterized by florid osseous metaplasia, has clinical features similar to those of conventional pilomatricoma but has a longer clinical history and firmer consistency. Microscopically, shadow cells are predominant along with extensive ossification, but minimal or no basaloid components are observed. Bone marrow formation and extramedullary hematopoiesis have also been reported to be associated with ossification.^[1,8,9]^ The tumor in our case showed typical microscopic features of the ossifying variant, which were similar to those observed in the late regressive stage; the tumor mainly contained shadow cell clusters and mature bony trabeculae without viable epithelial components. In addition, immunohistochemical analysis revealed that a few cells within the bone marrow were positive for antimyeloperoxidase antibodies, suggesting myeloblasts, but the marrow is considered nonhematopoietic. Ossifying variants represent old pilomatricomas. The long clinical history of our case suggests that these variants are old lesions. Ossifying variants are rarely found because most lesions are surgically removed before they become ossified. Conventional pilomatricomas are commonly brittle, but the surgical specimen of the ossifying variant has a stony hard consistency that is not shattered by ordinary physical forces.

Osseous metaplasia is a well-known aberrant ossification encountered in various neoplastic and non-neoplastic conditions. The pathogenesis of osseous metaplasia in ossifying variants remains unknown. However, considering that inflammatory infiltrate is replaced by calcification, ossification, and desmoplastic stroma in late-stage pilomatricomas, it is speculated that these histological changes may be a continuation of the foreign body reaction. Host responses to foreign materials typically include cellular and tissue responses: acute and chronic inflammation, granulation tissue formation, foreign body reaction, and stromal reactions such as fibrosis/fibrous capsule formation. Based on a series of host responses to foreign materials, stromal desmoplasia in the late regressive stage is considered a tissue reaction to keratinous materials containing shadow cells and an attempt to wall them off from the surrounding tissue. Likewise, we believe that osseous metaplasia in ossifying pilomatricomas is another tissue reaction to keratinous materials and a walling-off phenomenon to prevent exposure of surrounding tissues to keratinous materials. Thus, tissue reactions such as stromal desmoplasia and osseous metaplasia are considered biological phenomena that might be activated if cellular responses to foreign materials are recognized as insufficient. In particular, metaplastic ossification might be the next step in the desmoplastic reaction and the last host response to foreign materials in the body.

Aberrant ossification has been reported in other lesions associated with physiological or nonphysiological keratins: seborrheic keratosis,^[10]^ actinic keratosis,^[11]^ keratoacanthoma,^[12]^ acne vulgaris,^[13]^ epidermal cyst,^[14]^ trichilemmal cyst,^[15]^ desmoplastic trichoepithelioma,^[16]^ chondroid syringoma,^[17]^ and tumors histopathologically exhibiting shadow cells such as craniopharyngioma^[18]^ and calcifying odontogenic cyst.^[19]^ This phenomenon has also been observed in keratin-expressing tumors, including thymoma,^[20]^ sarcoma,^[21]^ and carcinomas such as basal cell carcinoma,^[22]^ squamous cell carcinoma,^[23]^ papillary thyroid carcinoma,^[24]^ and rectal carcinoma.^[25]^ Some of these lesions involve foreign body reactions, while others have the potential for reactions, although not reported in the literature. Keratin is a common substance that elicits foreign body reactions. Rupture of hair follicles and keratinous cysts allows previously sequestered keratin to come into direct contact with the surrounding tissue, where the keratin is subsequently recognized as foreign. The same is true for ingrown hair or nails.

In particular, keratin in hair or nails may be associated with ossifying lesions of melanocytic nevi,^[26,27]^ ingrown nails,^[28]^ and pyogenic granulomas.^[29,30]^ In melanocytic nevus with aberrant ossification, a foreign body reaction occurs in response to damaged hair follicles or free hair shafts.^[26,27]^ Reportedly, ossifying pyogenic granulomas often appear at or around the nail bed.^[29,30]^ Hence, the foreign body reactions observed in these lesions are considered to be associated with nail keratin. Similarly, this phenomenon may occur in conditions such as pseudofolliculitis barbae and acne keloidalis nuchae with potential for foreign body reactions due to ruptured or damaged hair follicles, although it has never been reported in the literature. Furthermore, aberrant ossification has been described in lesions such as tattooing^[31]^ and gout^[32]^ associated with other foreign materials.

Aberrant ossification has also been observed in conditions treated with prostheses such as breast augmentation,^[33]^ hip arthroplasty,^[34]^ hyoid suspension,^[35]^ temporomandibular joint reconstruction,^[36]^ and tibiofibular syndesmosis,^[37]^ and even in autoimmune diseases such as dermatomyositis,^[38]^ systemic sclerosis,^[39]^ and giant cell arteritis.^[40]^ Foreign body giant cells have been observed in these autoimmune diseases, although not simultaneously with ossification.^[41–43]^

Our comprehensive literature review revealed that aberrant ossification (metaplastic or heterotopic) occurred in various lesions or conditions with potential for foreign body reactions (Table 1). These findings suggest that aberrant ossification is closely related to foreign body reactions. However, aberrant ossification does not always occur in association with foreign body reactions. This phenomenon is believed to occur in response to foreign materials that evoke a strong immune reaction. Keratin is one of such foreign materials. In contrast, most biomaterials for medical use do not usually elicit a severe immune reaction in the body. Therefore, aberrant ossification is rarely observed in these circumstances. Despite the exposure of biological tissues to foreign materials, this phenomenon may or may not occur, depending on the type of foreign material, the individual's immune response, and duration of exposure.

**Table 1:** Aberrant ossification in lesions or conditions with potential for foreign body reactions.

Keratin	Nonkeratin	Prosthesis	Autoimmune
Seborrheic keratosis	Tattooing	Breast augmentation	Dermatomyositis
Actinic keratosis	Gout	Hip arthroplasty	Systemic sclerosis
Keratoacanthoma		Hyoid suspension	Giant cell arteritis
Acne vulgaris		TMJ reconstruction	
Ingrown nail		TF syndesmosis	
Melanocytic nevus			
Pyogenic granuloma			
Epidermal cyst			
Trichilemmal cyst			
Trichoepithelioma			
Chondroid syringoma			
Pilomatricoma			
Craniopharyngioma			
Calcifying odontogenic cyst			
Basal cell carcinoma			
Squamous cell carcinoma			
Thyroid carcinoma			
Rectal carcinoma			
Thymoma			
Synovial sarcoma			

TF = tibiofibular, TMJ = temporomandibular joint.

The mechanism of metaplastic ossification in pilomatricomas has not yet been elucidated. Some studies have reported that bone morphogenetic protein 2 in shadow cells plays a role in ectopic bone formation.^[44]^ Alternatively, oncostatin M produced by activated macrophages has been demonstrated to promote osteoblastic differentiation of precursor cells,^[45]^ and macrophage-derived osteopontin is suggested to play a significant role in the deposition of calcium phosphate in the shadow cell nests observed in pilomatricomas.^[46]^ We consider that foreign body reaction-induced macrophages play a more crucial role in metaplastic ossification by promoting osteoblastic differentiation and calcification rather than shadow cells.

In conclusion, we suggest that metaplastic ossification in ossifying pilomatricomas represents another feature of foreign body reaction to keratinous materials containing shadow cells in old lesions and a walling-off phenomenon to prevent exposure of surrounding tissues to keratinous materials. However, further studies are warranted to clarify the pathogenesis of metaplastic ossification in pilomatricomas.

## Author contributions

**Conceptualization:** Sang-Yeul Lee.

**Data curation:** Yeonjin Jeong.

**Methodology:** Kun-Yong Sung, Yeonjin Jeong.

**Visualization:** Seungkoo Lee.

**Writing – original draft:** Sang-Yeul Lee.

**Writing – review & editing:** Kun-Yong Sung, Seungkoo Lee.
